# International Migration, Refugees, and Spread of Tuberculosis in Brazil: Analysis of Clusters, Trends, and Associated Factors (2010–2021)

**DOI:** 10.3390/tropicalmed9040082

**Published:** 2024-04-11

**Authors:** Yan Mathias Alves, Thaís Zamboni Berra, Reginaldo Bazon Vaz Tavares, Marcela Antunes Paschoal Popolin, Fernanda Bruzadelli Paulino da Costa, Sonia Vivian de Jezus, Letícia Perticarrara Ferezin, Ariela Fehr Tártaro, Maria Del Pilar Serrano-Gallardo, Ione Carvalho Pinto, Ethel Leonor Noia Maciel, Ricardo Alexandre Arcêncio

**Affiliations:** 1 Ribeirão Preto College of Nursing, University of São Paulo, São Paulo 14040-902, Brazil; thais.berra@usp.br (T.Z.B.); reginaldobazon@usp.br (R.B.V.T.); marcelappopolin@gmail.com (M.A.P.P.); fernandabruzadelli@gmail.com (F.B.P.d.C.); lehferezin@usp.br (L.P.F.); ariela.fehr@gmail.com (A.F.T.); ricardo@eerp.usp.br (R.A.A.); 2 Nursing Department, Federal University of Tocantins, Palmas 77001-090, Brazil; 3 Instituto de Ciências da Saúde, Federal University of Mato Grosso, Sinop 78550-728, Brazil; 4 Nursing Department, Faculty of Medicine, Autonomous University of Madrid, 28029 Madrid, Spain; 5 Ministry of Health, Secretary of Health Surveillance and Environment, Brasília 70058-900, Brazil; ethel.maciel@gmail.com

**Keywords:** tuberculosis, immigration, spatial analysis

## Abstract

Background: International migration is a global phenomenon with significant implications on the health–disease process due to exposures along transit routes and local/destination epidemiological indicators. We aimed to analyze the transmission and spread of tuberculosis among international migrants and refugees from a spatiotemporal perspective and the associated factors. Method: This was an ecological study of cases of tuberculosis in international migrants in Brazil, between 2010 and 2021. Annual incidence rates were calculated and spatiotemporal scan techniques were used to identify municipalities at risk. Multiple logistic regression was used to identify factors associated with tuberculosis in international migrants. Results: A total of 4037 cases of tuberculosis were reported in Brazil in international migrants. Municipalities at risk for this event were identified using the spatiotemporal scan technique, and a cluster was identified with ITT: +52.01% and ETT: +25.60%. A higher probability of TB infection was identified in municipalities with a TB incidence rate >14.40 cases/100 inhabitants, population >11,042 inhabitants, Gini index >0.49, and illiteracy rate >13.12%. A lower probability was found in municipalities with average per capita household income >BRL 456.43. Conclusions: It is recommended that health authorities implement monitoring and rigorous follow-up in affected areas to ensure proper diagnosis and treatment completion for international migrants, preventing disease spread to other communities.

## 1. Introduction

International migration is a global social phenomenon that can have significant implications for the process of illness, especially for infectious diseases, due to pathogenic or environmental exposures along transit routes, transportation modes, and local and destination epidemiological indicators, thereby increasing the risk of acquiring certain diseases [1,2,3].

According to the United Nations High Commissioner for Refugees (UNHCR), international migrants are those who have chosen to live in another country and can safely return whenever they want. Refugees, on the other hand, have been forced to migrate due to issues related mainly to religious, political, racial, and social factors, and armed conflict, with serious and widespread human rights violations [4].

According to the World Migration Report [5], about 3.6% of the global population resides outside their country of origin, amounting to 281 million people. The situation is highlighted in Venezuela, which has had a significant impact on the migratory flows in Latin America and the Caribbean, and is one of the largest displacement and international migration crises [5]. In 2021, in Brazil, there were more than 151,000 legal international migrants and 29,000 requests for refuge, mostly from people coming from Venezuela, Angola, and Haiti, who entered mostly through the northern region of the country [6]. It is important to mention that the incidence of TB in Venezuela is equal to that of the native population of Brazil, i.e., 47 cases/100,000 inhabitants in both countries, making it difficult to establish where the transmission stream moves [7]. In Brazil, in 2021, 74,385 cases of TB were reported with an incidence of 34.9 TB cases per 100,000 [8].

Migrants and refugees are considered populations with a higher risk of developing tuberculosis (TB) [9,10] and other infectious diseases due to their precarious living conditions and overcrowding in refugee settlements, factors that potentially increase the risk of infection by Mycobacterium tuberculosis, the bacterium that causes the disease. Furthermore, it is important to mention that a relevant aspect that can contribute to the burden of infectious diseases in this population is the epidemiology of the country of destination for these immigrants, which can often present a higher incidence/prevalence rate than in the country of origin [11,12]. 

TB is known to be one of the most relevant global public health problems, being one of the leading causes of death by a single infectious agent in the world, causing 10.6 million cases and 1.4 million deaths in 2021 [13]. Thus, the impact of immigration on TB epidemiology is high [14], and the arrival of large groups can affect TB control efforts in destination countries, significantly increasing the burden of the disease and the cost of health services [10].

Most international migrants come from low-income countries that generally have a high burden of TB, imposing new challenges for global TB control and eradication. Likewise, most TB deaths occur in developing countries, including Brazil, which tends to be a frequent destination for international migrants, making the health–disease process a two-way street [15]. The main explanation for this reality is social inequality in these countries, where many people are not diagnosed correctly and/or in a timely manner by health services, in addition to difficulties in obtaining adequate treatment [15].

Brazil is the fifth largest country in terms of territorial extension, which is one of the challenges for disease control policies such as TB, combined with its vast land border, which represents 68% of the entire national territory. It is worth noting that the country shares borders with ten of the twelve countries in South America, with the exceptions of Chile and Ecuador. To the north, Brazil borders Suriname, Guyana, Venezuela, and French Guiana; to the northwest, it borders Colombia; to the west, it borders Peru and Bolivia; to the southwest, it borders Paraguay and Argentina; and to the south, it borders Uruguay [16]. This direct contact with several countries makes disease control even more complex and requires effective strategies that encompass border areas.

Brazil is an important destination for international migrants, with a significant growth in migratory waves in recent years. These waves have predominantly comprised people seeking work and better opportunities and living conditions, especially after COVID-19, which further exacerbated social inequities. Several studies aimed to study TB in international migrants in Brazil [15,17,18], but few have delved into this topic and sought to identify territories at risk and changes caused by the new coronavirus pandemic.

Given the above, this study sought to fill this knowledge gap and aimed to analyze the transmission and spread of TB among international migrants and refugees, from a space–time perspective, and the factors associated with the occurrence of tuberculosis.

## 2. Materials and Methods

### 2.1. Study Scenario

An ecological study was conducted using all 5570 Brazilian municipalities as the unit of analysis. Brazil is located in South America and is divided into five macro-regions (North, Northeast, Midwest, Southeast, and South), with 27 federative units. It has a territorial extension of 8,557,767 km² and a land border of 15,719 km, bordering ten South American countries, except Chile and Ecuador, and an estimated population of 208.4 million inhabitants [19].

#### 2.1.1. Study Population

All cases of TB diagnosed in international migrants reported between 2010 and 2021 in the Notifiable Diseases Information System (SINAN) were considered.

#### 2.1.2. Data Sources

Data encompassed TB cases diagnosed in international migrants in the Notifiable Diseases Information System (SINAN), which is the Brazilian information system responsible for recording and processing information on notifiable diseases throughout the country, including TB, and is one of the country’s main surveillance systems. These data were obtained through the Health Informatics Department of the Unified Health System (DATASUS), which is the IT department of the Brazilian Unified Health System (SUS). It is an entity belonging to the Ministry of Health, and is responsible for the collection, processing, storage, and dissemination of health information in Brazil. The information is in the public domain and can be accessed via the internet (https://datasus.saude.gov.br/ (accessed on 20 February 2023)).

### 2.2. Analysis Plan

#### 2.2.1. Time Series Analysis

Initially, annual incidence rates (2010 to 2021) of TB in international migrants were calculated, considering the number of TB cases reported in international migrants as the numerator and the population of international migrants residing per Brazilian municipality as the denominator, obtained through IBGE [19], and multiplied by the constant 100,000.

To verify the behavior of the time series over the study period and its temporal trend, the Seasonal Trend Decomposition using Loess (STL) decomposition method was used, which is based on locally weighted regression [20]. This analysis was performed using RStudio 2022.02.0+443 software through the forecast package [21].

After modeling the temporal trend of the event, using the same package mentioned, predictions were made up to the year 2030 to verify the behavior of the temporal trend of TB in international migrants in the coming years through the “auto.arima” command, which is based on the Box–Jenkins methodology or Autoregressive Integrated Moving Average model [22,23].

#### 2.2.2. Identification of Clusters

The study aimed to identify areas of risk for TB in international migrants in Brazil using the spatial analysis technique called scan statistics, developed by Kulldorff and Nagarwalla (1995) [24].

The identification of clusters was performed by positioning a circle of variable radius around the centroid of each Brazilian municipality (unit of analysis), and the number of observed and expected cases was calculated. This procedure was repeated until all municipalities’ centroids were tested, and when the observed value within the circle was higher or lower than expected, it was called a cluster [25].

The null hypothesis considers the non-existence of high- or low-risk clusters, i.e., the entire immigrant population has the same probability of contracting TB, regardless of their location. On the other hand, the alternative hypothesis assumes the existence of clusters where the population would have a higher or lower probability of contracting the disease [24].

In contrast to purely spatial scanning, which is based on circles, in space–time scanning, cylinders are created around each municipality’s centroid, where the circle’s base remains the same, and the cylinder’s height reflects the period of time considered in the cluster. Thus, incorporating time as a variable of interest allows the verification of the existence of clusters in a specific area that had a higher or lower proportion of cases than other analyzed areas during a specific period [25].

In addition to spatial and spatiotemporal analysis, the spatial variation in temporal trend (SVTT) technique was performed, which differs from the other techniques presented earlier by calculating the clusters’ temporal trends [26].

This analysis uses the same circles as purely spatial scanning, but SVTT does not seek to identify clusters with a high or low number of event occurrences. Instead, it verifies whether the event’s temporal trend is increasing or decreasing over time [25].

The temporal trend is calculated within and outside the scanning circle. We call the internal temporal trend (ITT) the change in the event’s temporal trend within a cluster, and the external temporal trend (ETT) the trend of all other areas that do not belong to this cluster. Therefore, what is statistically significant in this analysis is the temporal trends, rather than the cluster formation, as in spatial and spatiotemporal scanning [26,27].

Therefore, in SVTT, the null hypothesis considers no difference in temporal trends in the units of analysis, and the alternative hypothesis considers different temporal trends.

The parameters used in purely spatial, space–time, and SVTT scanning were a discrete Poisson model, no geographic overlap of clusters, circular-shaped clusters, and 999 replications in Monte Carlo simulation. Additionally, the size of the exposed population was stipulated by the Gini coefficient, where the number of cases was compared to the base population data. The expected number of cases in each municipality is proportional to the population size at risk [25,28].

In addition, the relative risk (RR) and confidence interval (95% CI) of each cluster were calculated, allowing the comparison of information in different areas, except for SVTT, as explained. The RR of the identified cluster may not be within the CI. Clusters with *p* < 0.05 were considered statistically significant.

The analyses were performed using SaTScan software version 9.3, and thematic maps were created using ArcGIS software version 10.5.

#### 2.2.3. Logistic Regression

To identify factors associated with the occurrence of TB in international migrants, multiple logistic regression was used. The variables selected to understand the phenomenon of interest were obtained based on the scientific literature and those collected by DATASUS (https://datasus.saude.gov.br/ (accessed on 20 February 2023)) and the Atlas of Human Development (http://www.atlasbrasil.org.br/ (accessed on 20 February 2023)). The Human Development Atlas in Brazil (Supplementary Material S1) is an official source from the Brazilian Government that provides information on the Municipal Human Development Index (IDHM) and over 200 other indicators related to demography, education, income, employment, housing, and vulnerability for Brazilian municipalities.

Before inserting the variables into the multiple regression model, these variables were dichotomized by their own medians, with 0 assigned to values below the median and 1 to values equal to or above the median. Thus, the dependent variable (DV) of the study was considered to be municipalities with reported cases of TB in international migrants, and the independent variables established were as follows: TB incidence rate in the general population; international migrant population; general population; average household per capita income; unemployment rate in individuals aged 16 or over; Gross Domestic Product (GDP) per capita; municipal GDP; households with sanitary facilities; Gini index; % of the population in households with piped water; % of people in urban households with garbage collection; % of people in households with electricity; % of the population living in households with density greater than two people per dormitory; % of people in households without electricity; % of people in households with walls that are not masonry or planed wood; % of people in households with inadequate water supply and sanitation; Municipal Human Development Index (MHDI); MHDI Education; and literacy rate.

Before conducting the logistic regression, an exploratory analysis of the selected variables was carried out regarding collinearity using the Variance Inflation Factor (VIF), where those with values greater than 10 would be removed from the statistical modeling; none of the analyzed variables exceeded this value, so all were included in the model [29].

The modeling was performed using the backward stepwise selection method, starting with a complete model (with all variables), removing one variable at a time, and checking the behavior of the model. The best model was considered the one that had the lowest Akaike Information Criterion (AIC) [29]. It should also be noted that, for the final model, odds ratios (ORs) were calculated with their respective 95% confidence interval (CI), using RStudio 2022.02.0+443 software. Variables were considered significant if they had a *p*-value < 0.05.

After all possibilities for analysis and selection of the final model were exhausted, Hosmer–Lemeshow, likelihood ratio, Cox–Snell, Nagelkerke, and McFadden tests were performed to validate the model. In addition, the predictive capacity and accuracy of the model were verified based on the area under the ROC (Receiver Operating Characteristic) curve and their respective 95% CI values [30]. The analyses and validation tests were performed using RStudio 2022.02.0+443 software.

## 3. Results

**Figure 1:**
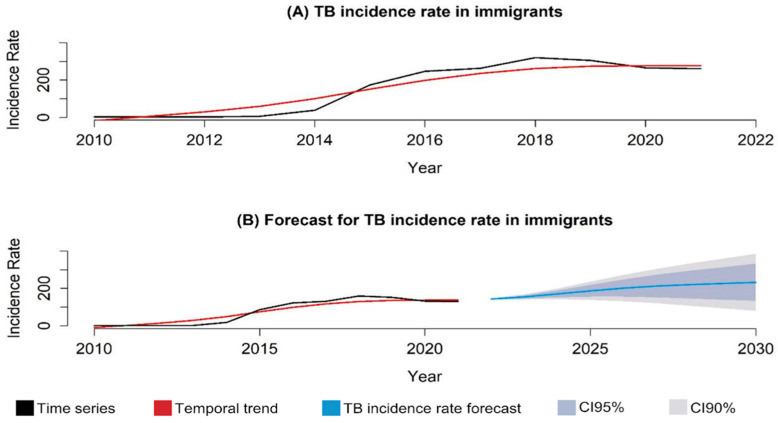
Temporal series and trend of tuberculosis cases in international migrants, Brazil (2010–2021). Legend: (**A**) temporal series and trend of tuberculosis incidence in international migrants and refugees reported in Brazil (2010–2021); (**B**) temporal trend and probability of tuberculosis incidence in international migrants and refugees in Brazil (2010–2021).

Between 2010 and 2021, 1,058,488 cases of TB were reported in Brazil, of which 4037 cases were in international migrants. Figure 1A shows the behavior of the temporal series of the disease in international migrants and its temporal trend over the study period, in which it is possible to observe that the temporal trend is increasing, similar to the trend observed in the forecast made up to 2030 (Figure 1B).

**Figure 2:**
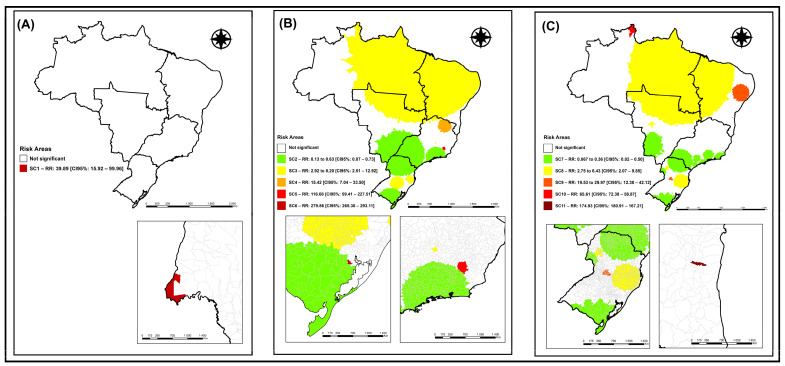
Spatial risk areas for the occurrence of tuberculosis in international migrants and refugees, Brazil (2010–2021). Legend: (**A**) spatial risk areas for tuberculosis occurrence in international migrants in Brazil, from 2010 to 2013; (**B**) spatial risk areas for tuberculosis occurrence in international migrants in Brazil, from 2014 to 2017; (**C**) spatial risk areas for tuberculosis occurrence in international migrants in Brazil, from 2018 to 2021.

In the spatial analysis and identification of risk and/or protection areas of the analyzed event, initially, to verify if there was a change and how the formation of purely spatial TB clusters in international migrants occurred, we opted to divide the analyzed period into four-year periods, with Figure 2A representing the period from 2010 to 2013, Figure 2B that from 2014 to 2017, and Figure 2C that from 2018 to 2021.

With the application of the purely spatial scanning technique, it was possible to identify a statistically significant cluster (*p* < 0.01) in the period corresponding to the years 2010 to 2013 (Figure 2A), corroborating the alternative hypothesis that there are areas in the municipality with a higher risk for the occurrence of TB in international migrants. The spatial cluster 1 (SC1) (Figure 2A), considered as a risk for the event, presented an RR:39.89 (95%CI: 15.92–99.96), formed by the municipality of Foz do Iguaçu (Paraná) in the Southern region of the country, with a population of 5747 international migrants, no expected cases, and seven observed cases.

The spatial cluster 2 (SC2) (Figure 2B) with RR:0.13 to 0.63 (95%CI: 0.07–0.73), was composed of 1566 municipalities in the Southern, Southeastern, and Central-West regions of Brazil, with a population of 202,301 international migrants, 725 expected cases, and 378 observed cases.

The spatial cluster 3 (SC3) (Figure 2B) presented RR:2.92 to 8.20 (95%CI: 2.51–12.92), was composed of 2421 municipalities encompassing all regions of the country, with a population of 25,631 international migrants, 92 expected cases, and 286 observed cases.

The spatial cluster 4 (SC4) (Figure 2B) with RR:15.42 (95%CI: 7.04–33.50), was composed of 70 municipalities in the Southeastern and Northeastern regions of Brazil, with a population of 109 international migrants, no expected cases, and six observed cases.

Spatial cluster 5 (SC5) (Figure 2B), with an RR:116.68 (95%CI: 59.41–227.51), was composed of 17 municipalities in the Southeast region of Brazil, with a population of 12 international migrants, no expected cases, and five observed cases.

Finally, spatial cluster 6 (SC6) (Figure 2B), with an RR:279.86 (95%CI: 265.30–293.11), was composed of the municipality of Mariana Pimentel (Rio Grande do Sul) in the Southern region of Brazil, with a population of four international migrants, no expected cases, and four observed cases.

Figure 2C represents the spatial clusters identified for the quadrennium from 2018 to 2021, where five clusters were identified; one was considered a protection cluster and four were considered risk clusters for TB in international migrants.

Spatial cluster 7 (SC7) (Figure 2C), considered a protection event, presented an RR ranging from 0.06 to 0.36 (95%CI: 0.02–0.50), composed of 919 municipalities in the Southern, Southeastern, and Central-Western regions of Brazil, with a population of 118,510 international migrants, 679 expected cases, and 160 observed cases.

Spatial cluster 8 (SC8) (Figure 2C) presented an RR ranging from 2.75 to 6.43 (95%CI: 2.07–9.85), composed of 1284 municipalities in the Southern, Central-Western, Northern, and Northeastern regions of Brazil, with a population of 16,286 international migrants, 93 expected cases, and 360 observed cases.

Spatial cluster 9 (SC9) (Figure 2C) with RR:19.53 to 29.97 (95%CI: 12.38–42.12), was composed of 265 municipalities in the South and Northeast regions of Brazil, with a population of 312 international migrants, 2 expected cases, and 44 observed cases.

Spatial cluster 10 (SC10) (Figure 2C) with RR:65.61 (95%CI: 72.38-58.87), was composed of four municipalities (Boa Vista, Normandia, Pacaraima, and Uiramuta) in the North region of Brazil, with a population of 866 international migrants, 5 expected cases, and 288 observed cases.

Finally, spatial cluster 11 (SC11) (Figure 2C) with RR:174.93 (95%CI:180.91-167.21), was formed by the municipality of Ubaitaba (Bahia), located in the Northeast region of Brazil, with a population of four international migrants, no expected cases, and four observed cases.

**Figure 3:**
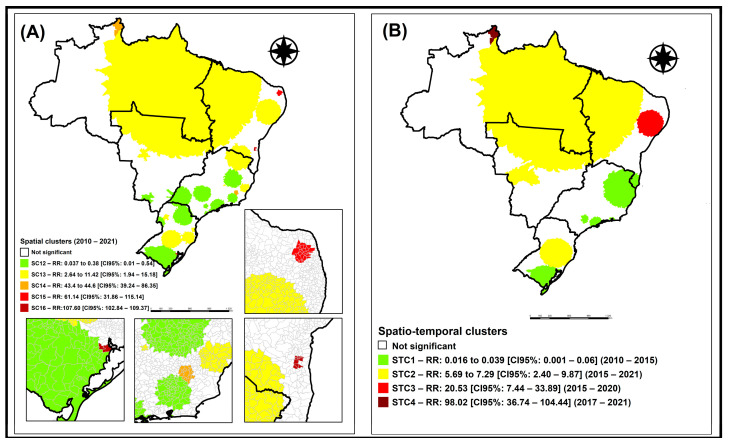
Spatial and space–time risk areas for the occurrence of tuberculosis in international migrants, Brazil (2010–2021). Legend: (**A**) spatial risk areas for the occurrence of tuberculosis in international migrants in Brazil, from 2010 to 2021; (**B**) spatiotemporal risk areas for the occurrence of tuberculosis in international migrants in Brazil, from 2010 to 2021.

Figure 3A represents the purely spatial scan analysis for the entire study period (2010 to 2021), in which five clusters were identified, one protective and four at risk for the occurrence of TB in international migrants.

Spatial cluster 1 (SC1) (Figure 3A), with RR:0.03 to 0.38 (95%CI: 0.01–15.18), was composed of 996 municipalities in the South, Midwest, and Southeast regions of Brazil, with a population of 110,695 international migrants, 1035 expected cases and 233 observed cases.

Spatial cluster 2 (SC2) (Figure 3A), with RR:2.64 to 11.42 (95%CI: 1.94–15.18), was composed of 2030 municipalities from all regions of Brazil, with a population of 19,937 international migrants, 183 expected cases, and 731 observed cases.

Spatial cluster 3 (SC3) (Figure 3A), with RR:43.4 to 44.6 (95%CI: 39.24–86.35), was composed of 21 municipalities in the Southeast region of Brazil, with a population of 878 international migrants, 8 expected cases, and 329 observed cases.

Spatial cluster 4 (SC4) (Figure 3A), with RR:61.14 (95%CI: 31.86–115.14), was composed of 28 municipalities in the Northeast region of Brazil, with a population of seven international migrants, no expected cases, and four observed cases.

Finally, spatial cluster 5 (SC5) (Figure 3A), with RR:107.60 (95%CI: 102.84–109.37), was composed of nine municipalities in the South and Northeast regions of Brazil, with a population of 11 international migrants, no expected cases, and 11 observed cases.

Figure 3B represents the space–time scan analysis for the entire study period (2010 to 2021), in which four clusters were identified, one of protection and three of risk for the occurrence of TB in international migrants.

Space–time cluster 1 (STC1) (Figure 3B), with RR:0.01 to 0.03 (95%CI: 0.00–0.06), was composed of 626 municipalities in the South, Southeast, and Northeast regions of Brazil, with a population of 6353 international migrants, 268 expected cases, and seven observed cases, during the period from 2010 to 2015.

Space–time cluster 2 (STC2) (Figure 3B), with RR:5.69 to 7.29 (95%CI: 2.40–9.87), was composed of 1675 municipalities in all regions of Brazil, with a population of 20,390 international migrants, 86 expected cases, and 528 observed cases, during the period from 2015 to 2021.

Space–time cluster 3 (STC3) (Figure 3B), with RR:20.53 (95%CI: 7.44–33.89), was composed of 321 municipalities in the Northeast region of Brazil, with a population of 378 international migrants, 2 expected cases, and 36 observed cases, during the period from 2015 to 2020.

Space–time cluster 4 (STC4) (Figure 3B), with RR:98.02 (95%CI: 36.74–104.44), was composed of four municipalities in the North region of Brazil (Normandia, Pacaraima, Uiramutã, and Boa Vista), with a population of 866 international migrants, 3 expected cases, and 306 observed cases.

**Figure 4:**
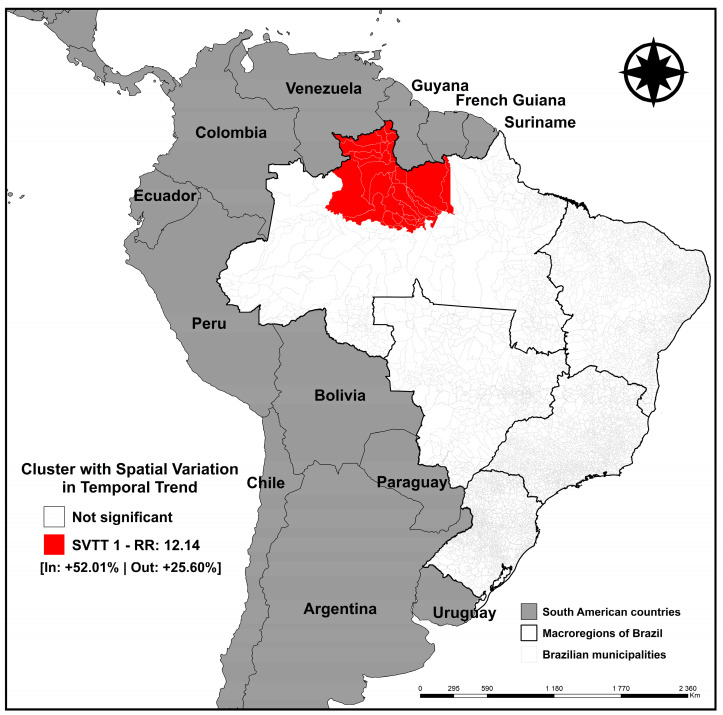
Spatial risk area with spatial variation in the temporal trend for the occurrence of tuberculosis in international migrants, Brazil (2010–2021).

Figure 4 represents the spatial scan analysis with spatiotemporal variation in the temporal trend for the entire study period (2010 to 2021), in which a high-risk cluster was identified for the occurrence of TB in international migrants, with RR:12.14, ITT:+52.01%, and ETT:+25.60%. This cluster was composed of 35 municipalities in the northern region of Brazil, with a population of 4723 international migrants, 44 expected cases, and 478 observed cases.

Through logistic regression (Table 1), it was possible to identify five variables associated with the occurrence of TB in international migrants, with four risk factors and one protective factor.

Thus, it was identified that international migrants are more likely to be infected with TB if they reside in municipalities with a TB incidence rate above 14.40 cases/100 inhabitants (OR: 3.08; 95%CI: 2.42–3.94); population above 11,042 inhabitants (OR: 3.23; 95%CI: 2.49–4.22); Gini index above 0.49 (OR:1.23; 95%CI: 1.01–1.52); and municipalities with illiteracy rate in individuals aged 15 or older above 13.12% (OR:1.77; 95%CI: 1.62–1.96).

As a protective factor, it was identified that international migrants residing in municipalities with an average per capita household income above BRL 456.43 are less likely to be infected with TB (OR:0.64; 95%CI: 0.47–0.87).

## 4. Discussion

The study aimed to analyze the transmission and spread of tuberculosis among international migrants and refugees from a spatiotemporal perspective, and the associated factors. The results showed that the trend of TB occurrence in the population of migrants and refugees is increasing, and the formation of risk clusters that require attention from municipal/state managers confirms that TB remains a problem, especially in the Northern and Northeastern regions of Brazil.

Using time series techniques, it was found that the temporal trend of TB in international migrants and refugees increased from 2010 to 2021, as well as in the forecast made until 2030, demonstrating that if no control measures or new disease mitigation policies are implemented, TB in international migrants and, consequently, in the Brazilian population, will continue to present increasingly concerning rates.

According to the OBMigra report (2019) [31], the nations with the most migrants to Brazil in the period before the COVID-19 pandemic were Venezuela, Paraguay, Bolivia, and Haiti, representing 53% of all registrations. According to the same report [30], from 2010 to 2019, the main Brazilian regions that received international migrants were the Southeast (44% of all registrations), mainly in the states of São Paulo and Rio de Janeiro; the South region, totaling 22% of registrations, distributed equally among the three states in the region (Paraná, Santa Catarina, and Rio Grande do Sul); and the North region, with 20% of all registrations, mainly concentrated in the states of Roraima (with emphasis on the capital Boa Vista and border regions with Venezuela) and Amazonas (the latter mainly near the state capital, Manaus). In 2019, among the international migrants registered in the North region, Roraima represented 38% of all registrations and had the highest number of annual registrations since 2010, an increase resulting from Venezuelan immigration to the region [31].

Corroborating these data, the municipalities with the highest incidence of TB cases in international migrants identified in the study belonged to the North, Northeast, and Southeast regions, with emphasis on the states of Roraima, Pernambuco, and Rio de Janeiro, respectively. These states historically belong to the group with the highest incidence and mortality rates of TB in the country. The state of Rio de Janeiro is an already consolidated destination for international migrants to Brazil, being the state in the Southeast region that receives the second most international migrants [6].

The Roraima region became central to the immigration flow, starting in 2016, because it was the preferred entry route for Venezuelans into the country [6], with this flow becoming even more exacerbated from 2018 due to the economic crisis affecting Venezuela. This corroborates the space–time cluster identified in Figure 3 (STC4), in which a cluster with RR of 98.02 (95% CI: 36.74–104.44) was found between 2017 and 2021, composed of the municipalities in the Northern region of Brazil, Normandia, Pacaraima, Uiramutã, and Boa Vista.

This same region was identified using spatial scan analysis of temporal trends (Figure 4), in which a risk cluster was found for the incidence of TB in international migrants composed of 35 municipalities in the Northern region of Brazil, presenting an internal temporal trend with twice the growth in the incidence of TB in international migrants when compared to the rest of the country.

In the latest report released by OBMigra [32], there has been a discrete increase in the registration of international migrant women in the country since 2011, and, from 2015, these numbers have grown exponentially, arousing interest in studies on the feminization process and the increase in the arrival of international migrant children and adolescents on Brazilian soil. In 2020, due to the COVID-19 pandemic, there was a decrease in this movement; however, in 2021, it started to grow again, totaling 151,155 international migrants, with 67,772 registrations of women, which is almost half of the international migrants registered in the country.

The increase in female migration in recent years, mainly from people coming from Haiti, can be explained by family reunification, in which men can migrate years earlier in search of job and education opportunities; once established in the country, they receive their relatives, especially their wives, companions, and children [33]. This is unlike what happens with most Venezuelan women, who are responsible for intensifying this process, and migrate along with their partners, children, and relatives [6].

It should be noted that the immigration status of women and children can lead to greater vulnerability to health issues due to gender problems and the higher risk of experiencing violence. In addition, children are more likely to develop respiratory infections due to malnutrition, and inadequate hygiene and housing conditions, during the migration process [9]. Nonetheless, men make up the majority of international migrants in the country and have a higher prevalence of TB compared to women [34,35].

Regarding the profile of this population, black and mixed-race international migrants stand out (62.4%), and those with a completed bachelor’s degree or higher education level (51.9%), followed by a completed high school level (27.1%). Between 2011 and 2021, the number of international migrant workers increased more than threefold, from 62.4 thousand to 188.0 thousand, respectively [32].

The dynamics of migratory flows to Brazil between 2010 and 2020 have placed the country back on the global stage of contemporary migration. The international economic crisis, which began in 2007 in the United States, and which also affected Europe and Japan, brought greater complexity to the Latin American migration phenomenon, having also caused growing human mobility between these countries and placing Brazil as a destination country [36]. It is worth noting that a factor that may have contributed to the country’s role as a migration destination in the regional scenario is its extensive border, which is shared with 10 out of the 12 countries in Latin America [16].

In the last decade (2011 to 2020), there has been an increase in migrants from the Global South, with a focus on Haitians, Venezuelans, Bolivians, Senegalese, Congolese, Angolans, Cubans, Bangladeshis, Syrians, and Pakistanis, among others. This is unlike the migratory flows from the late 19th century until the 1930s, in which the majority of international migrants were from the Global North (particularly Europe) [37].

Additionally, regarding this period (2011 to 2020), immigrations to Brazil have undergone several changes, among which the profile of international migrants who arrived in the country in relation to previous flows stands out, with a growing entry through the north border of the country, and an important labor insertion of international migrants in the South and Southeast regions. This has led to the need for changes in policies and regulation processes of international migrants, with the creation of Normative Resolutions by the National Immigration Council (CNIg) (RNs 27/2018, 97/2012, 126/2017), in the scope of the standardization of Haitian and Venezuelan international migrants, the new Migration Law of 2017 (Law 13.445), the creation of specific welcoming policies (Operation Acolhida in Boa Vista), and the process of interiorization of international migrants [32].

Regarding the relationship between migration and health in Latin American countries, a previous study [38] highlighted that immigration can increase the susceptibility of this population to infectious diseases, especially in migrant populations in situations of economic and social vulnerability. Lack of access to healthcare services and precarious living conditions can increase exposure to infectious diseases such as TB, hepatitis, and HIV/AIDS.

In addition, it is important to note that the COVID-19 pandemic has increased levels of poverty and income inequality worldwide, resulting in a significant increase in families, both national and international migrants, in situations of social vulnerability, thus directly impacting the rates of diseases related to social determinants of health, such as TB.

Observing the presented results, where more critical regions for the occurrence of TB in international migrants were identified, it is important to note that most of these territories already suffer from structural inequality and many of these international migrants face this reality when they arrive in Brazil, remaining in conditions of inequality and poverty [15].

The limitations of this study are related to its ecological design, meaning that the results obtained should not be extrapolated or interpreted at an individual level (ecological fallacy). The data used were obtained from secondary sources, which can generate a common bias in studies of this type due to recording errors, lack of information, or inconsistencies in data updates in all 5570 Brazilian municipalities, as well as the use of data from the last published census (2010). 

As another limitation of this study, we emphasize that including variables at the individual level to conduct the regression, such as age, gender, HIV, time since entering the country, or migration route, could enrich the study findings and, therefore, it is suggested that new studies with different approaches are possible.

It is also important to mention that in the present study, we observed a higher rate of new cases in migrants (109.2/100 thousand) than in the native population (49.5/100 thousand). Furthermore, because the TB screening policies in migrants in Brazil are different from those of screening in the general population, considering that migrants represent a population that is difficult to access, in addition to the fact that control of the migrant population in the country is difficult, rates can be overestimated.

However, the identification of critical areas is crucial for public health services and epidemiological surveillance, so it is necessary to concentrate efforts in these areas to combat the target event and avoid an increase in the number of cases. The time series analysis suggests a growing trend in the occurrence of TB in international migrants, which in itself raises an alert to managers. By using the spatial approach in conjunction with time series, it is possible to predict the worsening of the problem in the coming years if corrective measures are not taken, making it essential to adopt preventive measures immediately to identify cases, implement appropriate treatments, and minimize the suffering of this population.

It is also essential that public health policies be adapted and updated to meet the specific needs of migrant populations and that healthcare professionals be adequately trained to meet these demands, providing clear information and accessible language without any forms of discrimination.

## 5. Conclusions

This study used data from reported cases of tuberculosis from 2010 to 2021, in the pre-pandemic period and during the COVID-19 pandemic period. The impact of the COVID-19 pandemic on healthcare services in Brazil was to directly affect the decline in DOTS coverage, the discontinuation of active search for respiratory symptomatics, and education/awareness activities in the community. Because of these factors, the situation of TB in migrants increased, requiring greater attention from health authorities and civil society to achieve the goals of the End TB Strategy proposed by WHO.

Based on the results obtained, it is possible for health authorities to direct their initiatives to the affected areas, implementing rigorous monitoring and follow-up to ensure that patients complete their treatment and do not spread the disease to other locations or population groups. Due to the mobility of the migrant population, it is crucial to develop effective strategies to track these individuals throughout the Brazilian territory, in order to properly manage tuberculosis among international migrants.

## Figures and Tables

**Table 1:** Variables associated with the occurrence of tuberculosis in international migrants, Brazil (2010–2021).

Variable	Coefficient	*p*-Value	OR	95% CI
TB incidence rate (median: 14.40)	1.1262	<0.01 *	3.08	2.42–3.94
International migrant population	19.0827	0.94983	NA	NA
Municipality population (median: 11042)	1.1726	<0.01 *	3.23	2.49–4.22
Mean household income per capita (median: 456.43)	−0.4335	<0.01 *	0.64	0.47–0.87
Gini index 2010 (median: 0.49)	0.2125	0.04 *	1.23	1.01–1.52
Illiteracy rate—15 years or older (median: 13.12)	0.2529	0.02 *	1.77	1.62–1.96

R² = 2.34. OR, odds ratio value; CI, 95% confidence interval; *: *p* < 0.05 significant.

## Data Availability

The data presented in this study are available on request from the corresponding author on reasonable request.

## Supplementary Materials

The following supporting information can be downloaded at: https://www.mdpi.com/article/10.3390/tropicalmed9040082/s1, Document S1: Indicators of the Human Development Atlas in Brazil.

## References

[1] De Jezus S.V., Sales C.M.M., Rissino S.d.D., Mocelin H.J.S., Araújo M.P.d.S., Arcêncio R.A., Araújo V.M.S., Terena N.d.F.M., Freitas P.d.S.S., Maciel E.L.N. (2023). Prevalence of tuberculosis, COVID-19, chronic conditions and vulnerabilities among migrants and refugees: An electronic survey. Rev. Lat.-Am. Enferm..

[2] Abubakar I., Aldridge R.W., Devakumar D., Orcutt M., Burns R., Barreto M.L., Dhavan P., Fouad F.M., Groce N., Guo Y. (2018). The UCL–Lancet Commission on Migration and Health: The health of a world on the move. Lancet.

[3] Dhavan P., Dias H.M., Creswell J., Weil D. (2017). An overview of tuberculosis and migration. Int. J. Tuberc. Lung Dis..

[4] Alto Comissariado das Nações Unidas para Refugiados (ACNUR) (2018). Protegendo Refugiados no Brasil e no Mundo. https://www.acnur.org/portugues/wp-content/uploads/2018/02/Protegendo-Refugiados-no-Brasil-e-no-Mundo_ACNUR-2018.pdf.

[5] OIM, International Organization for Migration (2022). World Migration Report 2022.

[6] Cavalcanti L., Oliveira T., Silva B.G. (2021). Relatório Anual 2021—2011–2020: Uma Década de Desafios Para a Imigração e o Refúgio no Brasil.

[7] United Kingdom Health Security Agency (UKHSA) (2020). WHO Estimates of Tuberculosis Incidence by Country and Territory. https://www.gov.uk/government/publications/tuberculosis-tb-by-country-rates-per-100000-people/who-estimates-of-tuberculosis-incidence-by-country-and-territory-2020-accessible-text-version.

[8] Brasil (2023). Ministério da Saúde. Secretaria de Vigilância em Saúde e Ambiente|Ministério da Saúde. Boletim Epidemiológico. Tuberculose. https://www.gov.br/saude/pt-br/centrais-de-conteudo/publicacoes/boletins/epidemiologicos/especiais/2023/boletim-epidemiologico-de-tuberculose-numero-especial-mar.2023/view.

[9] Pavli A., Maltezou H. (2017). Health problems of newly arrived migrants and refugees in Europe. J. Travel Med..

[10] Meaza A., Tola H.H., Eshetu K., Mindaye T., Medhin G., Gumi B. (2022). Tuberculosis among refugees and migrant populations: Systematic review. PLoS ONE.

[11] Dasgupta K., Menzies D. (2005). Cost-effectiveness of tuberculosis control strategies among immigrants and refugees. Eur. Respir. J..

[12] Silva D.R., Mello F.C.d.Q., Johansen F.D.C., Centis R., D’Ambrosio L., Migliori G.B. (2023). Migration and medical screening for tuberculosis. J. Bras. Pneumol..

[13] World Health Organization (WHO) (2022). Global Tuberculosis Report. https://www.who.int/teams/global-tuberculosis-programme/tb-reports/global-tuberculosis-report-2022.

[14] MPareek M., Greenaway C., Noori T., Munoz J., Zenner D. (2016). The impact of migration on tuberculosis epidemiology and control in high-income countries: A review. BMC Med..

[15] Arcêncio R.A., Berra T.Z., Terena N.d.F.M., Rocha M.P., Alecrim T.F.d.A., Kihara F.M.d.S., Mascarello K.C., Sales C.M.M., Maciel E.L.N. (2021). Spatial clustering and temporal trend analysis of international migrants diagnosed with tuberculosis in Brazil. PLoS ONE.

[16] Instituto Brasileiro de Geografia e Estatística (IBGE) (2023). Municípios da Faixa de Fronteira [Internet]. https://www.ibge.gov.br/geociencias/organizacao-do-territorio/estrutura-territorial/24073-municipios-da-faixa-de-fronteira.html?=&t=sobre.

[17] Gonçalves D. (2019). Tuberculose em Imigrantes: Identificação e Análise das Características Associadas. Tese de Doutorado. Universidade de São Paulo. https://teses.usp.br/teses/disponiveis/17/17139/tde-12072019-105117/pt-br.php.

[18] Santo K.d.S.G.D., Paste A.A., Maturino H.S.d.A., Rocha V.O., Junior G.S.O., Coelho G.M., Oliveira M.S.S., Torres L.M. (2022). Desfecho de Pacientes com Tuberculose Pertencentes a Populações Vulneráveis no Brasil, em 2020. Braz. J. Infect. Dis..

[19] Instituto Brasileiro de Geografia e Estatística (2020). Brasil em Síntese [Internet]. https://www.ibge.gov.br/geociencias.

[20] Cleveland R., Cleveland W., McRee J.E. (1990). Seasonal-trend decomposition procedure based on LOESS. J. Off. Stat..

[21] Hyndman R., Athanasopoulos G., Bergmeir C., Caceres G., Chhay L., Kuroptev K., O’Hara-Wild M., Petropoulos F., Razbash S., Wang E. (2023). Forecasting: Forecasting Functions for Time Series and Linear Models. https://cran.r-project.org/web/packages/forecast/forecast.pdf.

[22] Box G.E., Jenkins G.M., Reinsel G.C., Ljung G.M. (2015). Time Series Analysis: Forecasting and Control.

[23] Camelo H.D.N., Lucio P.S., Junior J.B.V.L., de Carvalho P.C.M. (2017). Métodos de previsão de séries temporais e modelagem híbrida ambos aplicados em médias mensais de velocidade do vento para regiões do nordeste do Brasil. Rev. Bras. Meteorol..

[24] Kulldorff M., Nagarwalla N. (1995). Spatial disease clusters: Detection and inference. Stat. Med..

[25] Kulldorff M. (2015). SaTScan User Guide V9.4. https://edisciplinas.usp.br/pluginfile.php/7199540/mod_resource/content/2/SaTScan_TM_Manual_do_Usuario_v9.4_Portugues_2016_05.pdf.

[26] Moraga P., Kulldorff M. (2016). Detection of spatial variations in temporal trends with a quadratic function. Stat. Methods Med Res..

[27] Jaisankar R., Kesavan J. (2019). A study on spatial variations in temporal trends of dengue incidences in Tamil Nadu, India. Int. J. Sci. Technol. Res..

[28] Han J., Zhu L., Kulldorff M., Hostovich S., Stinchcomb D.G., Tatalovich Z., Lewis D.R., Feuer E.J. (2016). Using Gini coefficient to determining optimal cluster reporting sizes for spatial scan statistics. J. Health Geogr..

[29] Zuur A.F., Ieno E.N., Elphick C.S. (2010). A protocol for data exploration to avoid common statistical problems. Methods Ecol. Evol..

[30] Šimundić A.M. (2009). Medidas de acurácia diagnóstica: Definições básicas. Electron. J. Int. Fed. Clin. Chem. Lab. Med..

[31] Cavalcanti L., Oliveira T., Macedo M. (2019). Imigração e Refúgio no Brasil. Relatório Anual 2019.

[32] Cavalcanti L., Oliveira T., Silva B.G. (2022). Relatório Anual OBMigra 2022.

[33] Mamed L.H., De Lima E.O. (2015). Trabalho, precarização e migração: O processo de recrutamento de haitianos na Amazônia acreana pela agroindústria brasileira. Novos Cad. NAEA.

[34] Barreto-Duarte B., Araújo-Pereira M., Nogueira B.M.F., Sobral L., Rodrigues M.M.S., Queiroz A.T.L., Rocha M.S., Nascimento V., Souza A.B., Cordeiro-Santos M. (2021). Tuberculosis Burden and Determinants of Treatment Outcomes According to Age in Brazil: A Nationwide Study of 896,314 Cases Reported Between 2010 and 2019. Front. Med..

[35] Hertz D., Schneider B. (2018). Sex differences in tuberculosis. Semin. Immunopathol..

[36] Cavalcanti L., Tonhati T., Dutra D., De Oliveira M. (2016). A Imigração Haitiana no Brasil: Características Sociodemográficas e Laborais na Região Sul e no Distrito Federal.

[37] Gómez C., Herrera G. (2022). State and ‘Mixed Migrations’: Migration Policies towards Haitians, Colombians and Venezuelans in Ecuador. Migration in South America.

[38] Díaz-Quijano F.A., Rodríguez-Morales A.J., Waldman E.A. (2018). Migration, public health and infectious diseases in South America. PLoS ONE.

