# Supplementary material for: International Migration, Refugees, and Spread of Tuberculosis in Brazil: Analysis of Clusters, Trends, and Associated Factors (2010–2021)
Source: Trop Med Infect Dis. 2024 Apr 11;9(4):82. doi: 10.3390/tropicalmed9040082 (PMC11053982; doi:10.3390/tropicalmed9040082)
Supplement: Supplementary file 1 [file tropicalmed-09-00082-s001.zip › tropicalmed-2929846-supplementary.pdf]

Indicators of the Human Development Atlas in Brazil

(http://www.atlasbrasil.org.br/)

Human Development

National Household Sample Survey (PNAD)

- Education Index - Education HDI
- School attendance index - Education HDI
- Education HDI ***
- Human Development Index (HDI)***
- Income HDI
- Longevity HDI
- Inequality-adjusted HDI
- Inequality-adjusted Longevity HDI
- Inequality-adjusted Education HDI
- Inequality-adjusted Income HDI
- Inequality-adjusted HDI - Loss due to inequality
- Inequality-adjusted Longevity HDI - Loss due to inequality
- Inequality-adjusted Education HDI - Loss due to inequality
- Inequality-adjusted Income HDI - Loss due to inequality
- Life expectancy at birth
- Per capita income***

Census

- Education HDI
- Education Index - Education HDI
- School attendance index - Education HDI
- HDI
- Income HDI
- Longevity HDI
- Life expectancy at birth
- Per capita income

Education

- National Household Sample Survey (PNAD)
- Illiteracy rate - 15 years old and over ***
- Illiteracy rate - 25 years old and over ***
- Illiteracy rate - 18 years old and over ***
- % of 5 to 6-year-olds in school
- % of 15 to 17-year-olds in school
- % of 6 to 14-year-olds in school
- % of 18 to 24-year-olds in school

- % of 6 to 14-year-olds in elementary school with 2 or more years of delay in grade
- % of 6 to 17-year-olds in basic education with 2 or more years of delay in grade
- % of 25 years old and over with complete higher education
- % of 15 to 17-year-olds with complete elementary education
- % of 18 to 24-year-olds with complete elementary education
- % of 18 years old and over with complete elementary education
- % of 25 years old and over with complete elementary education
- % of 18 to 20-year-olds with complete high school education
- % of 25 years old and over with complete high school education
- Average years of schooling
- Net enrollment rate in basic education
- Net enrollment rate in elementary education
- Net enrollment rate in high school education
- Net enrollment rate in higher education
- % of 11 to 13-year-olds in the final years of elementary school or with complete elementary education

Census

- Illiteracy rate - 11 to 14 years old
- Illiteracy rate - 15 years old and over
- Illiteracy rate - 15 to 17 years old
- Illiteracy rate - 18 years old and over
- Illiteracy rate - 18 to 24 years old
- Illiteracy rate - 25 years old and over
- Illiteracy rate - 25 to 29 years old
- % of 0 to 5-year-olds in school
- % of 5 to 6-year-olds in school
- % of 6 to 14-year-olds in school
- % of 6 to 17-year-olds in school
- % of 15 to 17-year-olds in school
- % of 18 to 24-year-olds in school
- % of 25 to 29-year-olds in school
- % of 6 to 14-year-olds in elementary school with 2 or more years of delay in grade
- % of 15 to 17-year-olds attending elementary school
- % of 6 to 17-year-olds in basic education with 2 or more years of delay in grade
- % of 18 to 24-year-olds attending elementary school
- % of 18 to 24-year-olds attending elementary school
- % of 18 years old and over with complete elementary education
- Expected years of schooling at 18 years old
- % of 15 to 17-year-olds with complete elementary education
- % of 18 to 24-year-olds with complete elementary education
- % of 25 years old and over with complete elementary education
- % of 18 to 20-year-olds with complete high school education
- % of 18 to 24-year-olds with complete high school education
- % of 18 years old and over with complete high school education

- % of 25 years old and over with complete higher education
- % of 25 years old and over with complete high school education
- Gross enrollment rate in preschool
- Gross enrollment rate in elementary education
- Gross enrollment rate in high school education
- Gross enrollment rate in basic education
- Gross enrollment rate in higher education
- Net enrollment rate in preschool
- Net enrollment rate in elementary education
- Net enrollment rate in high school education
- Net enrollment rate in basic education
- Net enrollment rate in higher education
- % of 11 to 13-year-olds in the final years of elementary school or with complete elementary education
- % of 12 to 14-year-olds in the final years of elementary school or with complete elementary education

School Census

- % of enrollments in private schools in high school
- % of enrollments in public schools in elementary education
- % of enrollments in public schools in high school
- % of enrollments in private schools in elementary education
- Age-Grade distortion rate in elementary education in public schools
- Age-Grade distortion rate in elementary education
- Age-Grade distortion rate in high school in private schools
- Age-Grade distortion rate in high school in public schools
- Age-Grade distortion rate in high school
- Age-Grade distortion rate in elementary education in private schools
- Dropout rate in elementary education
- Dropout rate in high school
- Dropout rate in elementary education in private schools
- Dropout rate in high school in private schools
- Dropout rate in elementary education in public schools
- Dropout rate in high school in public schools
- Initial Years of Elementary Education Development Index
- Final Years of Elementary Education Development Index
- % of elementary school students in schools with computer labs
- % of high school students in schools with computer labs
- % of elementary school students in schools with internet access
- % of high school teachers with adequate training
- % of elementary school teachers in public schools with adequate training
- % of high school teachers in public schools with adequate training
- % of elementary school teachers in private schools with adequate training
- % of high school teachers in private schools with adequate training
- % of high school students in schools with internet access

- % of elementary school teachers with adequate training

Housing

National Sanitation Information System (SNIS)

- % of urban population served by regular household waste collection services ***
- % of urban population living in households connected to the water supply network ***
- Existence of selective waste collection
- % of urban population living in households connected to the sewage system ***
- % of treated sewage

Census

- % of population in households with piped water
- % of population living in households with bathroom and piped water ***
- % of people in urban households with garbage collection ***
- % of people in households with electricity ***
- % of population living in households with a density of more than 2 people per bedroom ***
- % of people in households with walls that are not masonry or treated wood ***

Environment

MapBiomas

- % of natural vegetation cover

National Institute for Space Research (INPE)

- Concentration of hotspots

Political Participation

Superior Electoral Court (TSE)

- % of female state deputies
- % of female federal deputies
- % of female governors
- % of female mayors
- % of female senators
- % of female city councilors

Population

Census

- Population in vulnerable households with elderly individuals
- Population of female heads of households with at least one child under 15 years old
- Population aged 15 to 24 vulnerable to poverty
- Employed population vulnerable to poverty commuting daily from work to home
- Infant mortality
- Under-5 mortality
- Probability of survival until age 40
- Probability of survival until age 60
- Dependency ratio
- Total fertility rate
- Aging rate
- Population aged 0 to 1 year
- Population aged 18 years and older
- Population aged 1 to 3 years
- Total population
- Rural population
- Urban population
- Male population aged 0 to 4 years
- Male population aged 5 to 9 years
- Male population aged 10 to 14 years
- Male population aged 15 to 19 years
- Male population aged 20 to 24 years
- Male population aged 25 to 29 years
- Male population aged 30 to 34 years
- Male population aged 35 to 39 years
- Male population aged 40 to 44 years
- Male population aged 45 to 49 years
- Male population aged 50 to 54 years
- Male population aged 55 to 59 years
- Male population aged 60 to 64 years
- Male population aged 65 to 69 years
- Male population aged 70 to 74 years
- Male population aged 75 to 79 years
- Male population aged 80 years and older
- Female population aged 0 to 4 years
- Female population aged 5 to 9 years
- Female population aged 10 to 14 years
- Female population aged 15 to 19 years
- Female population aged 20 to 24 years
- Female population aged 25 to 29 years
- Female population aged 30 to 34 years
- Female population aged 35 to 39 years
- Female population aged 40 to 44 years
- Female population aged 45 to 49 years
- Female population aged 50 to 54 years
- Female population aged 55 to 59 years

- Female population aged 60 to 64 years
- Female population aged 65 to 69 years
- Female population aged 70 to 74 years
- Female population aged 75 to 79 years
- Female population aged 80 years and older
- Total male population
- Total female population
- Population aged 4 years
- Population aged 5 years
- Population aged 6 years
- Population aged 6 to 10 years
- Population aged 10 years and older
- Population aged 10 to 14 years
- Population aged 11 to 14 years
- Population aged 15 years and older
- Population aged 15 to 17 years
- Population aged 15 to 17 years
- Population aged 18 years and older
- Population aged 18 to 24 years
- Population aged 25 years and older
- Population aged 65 years and older
- Total population in permanent private households
- Total population in permanent private households, excluding those with zero income
- Women aged 12 to 14 years
- Women aged 15 to 17 years
- Women aged 15 years and older
- Women aged 25 years and older
- Economically active population aged 10 years and older
- Economically active population aged 10 to 14 years
- Economically active population aged 15 to 17 years
- Economically active population aged 18 years and older

National Household Sample Survey (PNAD)

- Infant mortality
- Aging rate
- Dependency ratio
- Total population
- Total population in permanent private households, excluding those with zero income
- Population aged 6 to 17 years
- Population aged 6 to 14 years
- Population aged 65 years and older
- Population aged 5 to 6 years
- Population aged 25 years and older
- Population aged 18 years and older
- Population aged 18 to 24 years

- Population aged 18 to 20 years
- Population aged 15 years and older
- Population aged 15 to 17 years
- Population aged 11 to 13 years
- Total population in permanent private households

Instituto Brasileiro de Geografia e Estatística (IBGE)

- Total population ***
- Population ***

Income

Annual List of Social Information (RAIS)

- Gross Domestic Product per capita ***
- Municipal Gross Domestic Product ***
- Service Sector's Share in Value Added
- Public Administration's Share in Value Added
- Industry's Share in Value Added
- Agriculture's Share in Value Added
- Value Added per capita
- Per capita transfer from Continuous Cash Benefit
- Per capita transfer from Bolsa Família
- Average income in the formal sector

National Household Sample Survey (PNAD)

- Theil-L Index
- Gini Index ***
- % of population vulnerable to poverty
- % of poor population
- % of extremely poor population
- % of income from labor earnings
- Per capita income of the population vulnerable to poverty
- Per capita income of the poor population
- Per capita income of the extremely poor population
- Per capita income, excluding zero income
- Average per capita income of the top 10%
- Average per capita income of the top 20%
- Average per capita income of the fourth quintile
- Average per capita income of the third quintile
- Average per capita income of the second quintile
- Average per capita income of the bottom quintile

- Ratio 20% richest / 40% poorest
- Ratio 10% richest / 40% poorest

Single Registration

- % of individuals registered in the Single Registry receiving Bolsa Família
- % of population vulnerable to poverty in the Single Registry post-Bolsa Família
- % of poor population in the Single Registry post-Bolsa Família
- % of extremely poor population in the Single Registry post-Bolsa Família

Census

- Per capita income, excluding zero income
- % of income from labor earnings
- Average per capita income of the bottom quintile
- Maximum per capita household income of the bottom quintile
- Average per capita income of the second quintile
- Maximum per capita household income of the second quintile
- Average per capita income of the third quintile
- Maximum per capita household income of the third quintile
- Average per capita income of the fourth quintile
- Maximum per capita household income of the fourth quintile
- Average per capita income of the top quintile
- Average per capita income of the top 10%
- Minimum per capita household income of the top 10%
- % of extremely poor population
- % of poor population
- % of population vulnerable to poverty
- % of children under 14 years old extremely poor
- % of children poor
- % of children vulnerable to poverty
- Per capita income of the extremely poor population
- Per capita income of the poor population
- Per capita income of the population vulnerable to poverty
- % of individuals belonging to the bottom quintile of the distribution of individuals by per capita household income
- % of individuals belonging to the bottom two quintiles of the distribution of individuals by per capita household income
- % of individuals belonging to the bottom three quintiles of the distribution of individuals by per capita household income
- % of individuals belonging to the bottom four quintiles of the distribution of individuals by per capita household income
- 10% richest
- % of individuals belonging to the top quintile of the distribution of individuals by per capita household income
- Ratio 20% richest / 40% poorest

- Ratio 10% richest / 40% poorest
- Gini Index
- Theil-L Index
- Average earnings of the employed
- % of employed individuals with no income
- % of employed individuals earning up to 1 minimum wage
- % of employed individuals earning up to 2 minimum wages
- % of employed individuals earning up to 3 minimum wages
- % of employed individuals earning up to 5 minimum wages
- Theil-L Index of labor earnings - 18 years and older

Health

Department of Informatics of the Unified Health System (DATASUS)

- % of hospitalizations due to diseases related to inadequate environmental sanitation
- % of hospitalizations due to conditions sensitive to primary care
- Gross mortality rate
- % of people covered by supplementary health plans
- Incidence rate of AIDS
- TB incidence rate ***
- Mortality rate due to aggression
- Mortality rate due to traffic accidents
- Mortality rate of women due to breast cancer
- Mortality rate due to prostate cancer
- Mortality rate due to non-communicable diseases
- Homicide mortality rate
- Infant mortality rate
- Suicide mortality rate
- % of live births with at least seven prenatal care visits
- % of live births with low birth weight
- Maternal mortality rate

Work

Census

- Activity rate - 10 years and older
- Activity rate - 10 to 14 years old
- Activity rate - 15 to 17 years old
- Activity rate - 18 years and older
- Activity rate - 18 to 24 years old
- Activity rate - 25 to 29 years old
- Unemployment rate - 10 years and older
- Unemployment rate - 10 to 14 years old
- Unemployment rate - 15 to 17 years old ***

- Unemployment rate - 18 years and older ***
- Unemployment rate - 18 to 24 years old ***
- Unemployment rate - 25 to 29 years old ***
- % of employed individuals 18 years and older with formal employment
- % of employed individuals 18 years and older without formal employment
- % of employed individuals 18 years and older in the public sector
- % of employed individuals 18 years and older self-employed
- % of employed individuals 18 years and older employers
- Degree of formalization of employed individuals - 18 years and older
- % of employed individuals with complete elementary education
- % of employed individuals with complete high school education
- % of employed individuals with complete higher education
- % of employed individuals in the agricultural sector
- % of employed individuals in the mineral extraction sector
- % of employed individuals in the manufacturing industry
- % of employed individuals in public utility industrial services
- % of employed individuals in the construction sector
- % of employed individuals in the trade sector
- % of employed individuals in the services sector

Vulnerability

Census

- % of people in poverty-vulnerable households spending more than one hour commuting to work out of the total population of vulnerable and employed individuals who commute daily
- % of single mothers without complete elementary education and with at least one child under 15 years old, out of the total single mothers with minor children
- % of vulnerable people dependent on elderly individuals, out of the total population in vulnerable households with elderly residents
- % of 15 to 24-year-olds not studying, not working, and vulnerable, within the vulnerable population in this age group
- % of girls aged 10 to 17 who have had children
- % of single mothers without complete elementary education and with at least one child under 15 years old
- % of children living in households where none of the residents have completed elementary education
- % of children aged 0 to 5 not attending school
- % of children aged 6 to 14 not attending school
- % of people in households where no resident has completed elementary education
- % of 15 to 24-year-olds not studying or working in poverty-vulnerable households
- % of individuals aged 18 or older without complete elementary education and in informal employment
- % of people in poverty-vulnerable households where no one has completed elementary education
- % of people in poverty-vulnerable households dependent on elderly individuals

- % of people in households without electricity ***
- % of people in households with inadequate water supply and sanitation ***
- % of people in poverty-vulnerable households spending more than one hour commuting to work

Department of Informatics of the Unified Health System (DATASUS)

- % of girls aged 10 to 14 who have had children
- % of teenagers aged 15 to 17 who have had children

Single Registry

- % of illiterates aged 15 or older in the Single Registry
- % of individuals enrolled in the Single Registry without adequate water supply
- % of individuals enrolled in the Single Registry without adequate sanitation
- % of individuals enrolled in the Single Registry without proper garbage collection
- % of individuals enrolled in the Single Registry without adequate water supply, sanitation, and garbage collection
- % of individuals enrolled in the Single Registry without adequate water supply, sanitation, and garbage collection

*** Indicators of the Human Development Atlas in Brazil used in the study
